# Potential Nitrogen-Based Heterocyclic Compounds for Treating Infectious Diseases: A Literature Review

**DOI:** 10.3390/antibiotics11121750

**Published:** 2022-12-03

**Authors:** Mohammad Aatif, Muhammad Asam Raza, Khadija Javed, Swah Mohd. Nashre-ul-Islam, Mohd Farhan, Mir Waqas Alam

**Affiliations:** 1 Department of Public Health, College of Applied Medical Sciences, King Faisal University, Al-Ahsa 31982, Saudi Arabia; 2 Department of Chemistry, Hafiz Hayat Campus, University of Gujrat, Gujrat 50700, Pakistan; 3 Department of Chemistry, Mangaldai College, Assam 784125, India; 4 Department of Basic Sciences, Preparatory Year Deanship, King Faisal University, Al-Ahsa 31982, Saudi Arabia; 5 Department of Physics, College of Science, King Faisal University, Al-Ahsa 31982, Saudi Arabia

**Keywords:** heterocyclic compound, medicinal chemistry, infectious disease, organic synthesis, in-vitro studies

## Abstract

Heterocyclic compounds are considered as one of the major and most diverse family of organic compounds. Nowadays, the demand for these compounds is increasing day-by-day due to their enormous synthetic and biological applications. These heterocyclic compounds have unique antibacterial activity against various Gram-positive and Gram-negative bacterial strains. This review covers the antibacterial activity of different heterocyclic compounds with nitrogen moiety. Some of the derivatives of these compounds show excellent antibacterial activity, while others show reasonable activity against bacterial strains. This review paper aims to bring and discuss the detailed information on the antibacterial activity of various nitrogen-based heterocyclic compounds. It will be helpful for the future evolution of diseases to synthesize new and effective drug molecules.

## 1. Introduction

Bacteria cause different infectious diseases, which can have a compelling effect on health and become the main reason for the bulk of hospital-acquired diseases responsible for a huge mortality rate and immense responsibility on healthcare systems. Various advanced malignant bacterial forms have appeared, with distinctive levels of resistance against a therapeutic agent. The current infections may be due to *Escherichia coli,* multidrug-resistant tuberculosis [1], and methicillin-resistant *Staphylococcus aureus* [2], etc. So, there is a persuasive demand for the development of new and more competent drugs across both drug-sensitive and drug-resistant micro-organisms. In recent times, for the synthesis of advanced drugs, greater concentration has been paid to the progress of heterocyclic scaffolds [3]. Among the heterocyclic compounds, the ones containing nitrogen atom(s) are of huge interest, since they constitute a significant class of distinct non-natural and natural products [4]. With the increase in the synthesis of new drugs from different medicinal experts, the resistance against these drugs by the microbes is also increasing with good pace. Many microbes are showing resistance against 2nd and 3rd generation antibiotics. So, in order to fight with resistance there is a need for the synthesis of new drugs with fruitful antimicrobial potential. The main objective of this review is to provide a literature survey to the readers about the synthesis and anti-infectious potential of different nitrogen-based heterocyclic compounds.

### 1.1. Heterocyclic Compounds

Heterocyclic compounds are cyclic compounds which contain the atoms of two discrete elements as representative of their ring(s). They belong to one of the larger classes of organic compounds and also appear more valuable in different fields of chemistry [5]. These compounds encompass a higher number of drugs, most biomass, nucleic acids, synthetic dyes, and numerous natural products such as alkaloids, herbicides, vitamins, antibiotics, hormones, and pharmaceutics [6]. Heterocyclic compounds have tremendous importance for both organic and medical chemists, and the synthesis of such molecules always remains a challenge from both industrial and academic aspects [7]. Heterocyclic systems with different structures display various biologic activities because of diversities in their molecular structures [8]. All these systems have been used for the composition of unusual drugs due to their unique physicochemical properties. Furthermore, the systems of these compounds have been very attractive in recent times because of both sulfur and nitrogen atoms present in them [9]. In therapeutic perseverance, to deal with the various types of bacterial and fungal infections along with the analysis of cancer, gastric ulcers, and many other diseases, these compounds have been extensively utilized [10].

### 1.2. Application of Heterocyclic Compounds

**Figure 1:**
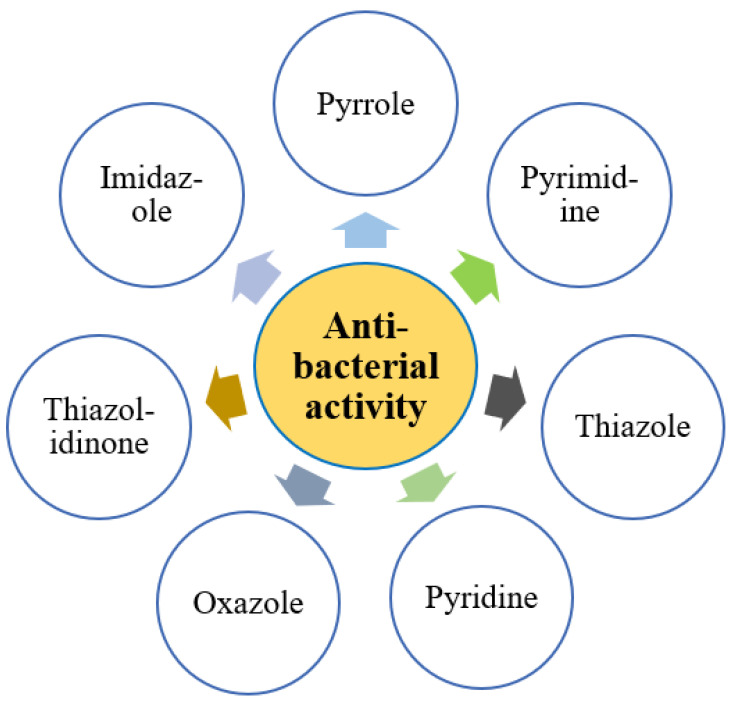
Literature survey of antibacterial activity of nitrogen-based heterocyclic compounds.

In biochemical processes, heterocyclic compounds play a significant role due to the existence of aromatic heterocycles in the side groups of the utmost typical and fundamental constituents of all living cells [11]. These heterocyclic compounds, along with organic compounds with their biological activities, are utilized as drugs in many veterinary and human medicines, and are also used as pesticides and insecticides in agriculture. Many marketed drugs show the presence of these chemical rings (Figure 1). The existence of these rings exhibited that such types of drugs have pharmaceutical properties and at the same time can present a platform for diverse types of pharmacophoric groups, which can interact with receptors [12].

The researchers’ interest has been primarily on nitrogen and sulfur-containing heterocyclic compounds through the advancement of organic synthesis. Most of them are tetrazoles, triazoles, fused thiazoles, oxadiazoles and thiadiazoles, which are constitutional subunits of different biologically active compounds [13]. Derivatives of thiadiazole possess biological activity owing to the vigorous aromaticity of the ring system, which further helps in acquiring considerable *in vivo* stability and also in reducing the toxicity of higher vertebrates, along with humans [11]. Among different heterocycles, pyranopyrimidines and derivatives of benzimidazole are an essential type of heterocyclic pharmaceutical. Such compounds display intriguing biological properties, such as anti-inflammatory, anti-fungal, anti-microbial, anti-phlogistic, antibacterial, anti-thrombotic and anti-genotoxic activity [14]. Oxazole is an oxygen-containing heterocyclic compound, which plays a very pivotal role in the formation of such numerous biologically effective drugs as anti-inflammatory, anti-cancer, anti-depressant, anti-microbial, anti-obesity, anti-diabetic, and analgesic [15]. Moreover, for several years, interest in the derivatives of pyrrole as anti-microbial agents has impelled the formation and antimicrobial estimation of hundreds of distinctive molecules such as monodeoxypyoluteorin and derivatives of 2-(2’-hydroxybenzoyl) pyrrole bromine [16].

Thus, analysts have always been searching for such unique synthetic methodologies that would help us to have an approach in more economical, potent, and selective ways on the heterocyclic cores. Correspondingly, in the literature, various efficient illustrations are available. Cycloaddition reactions are one of the most competent and straightforward approaches to access the cores of different heterocyclic compounds [17]. These types of reactions not only occur with excellent atom economy but also allow the simultaneous manufacturing of two bonds; albeit this method has some limitations [18].

## 2. Antibacterial Activity of Heterocyclic Compounds

### 2.1. Synthesis and Antibacterial Activity of the Pyrrole

**Scheme 1:**
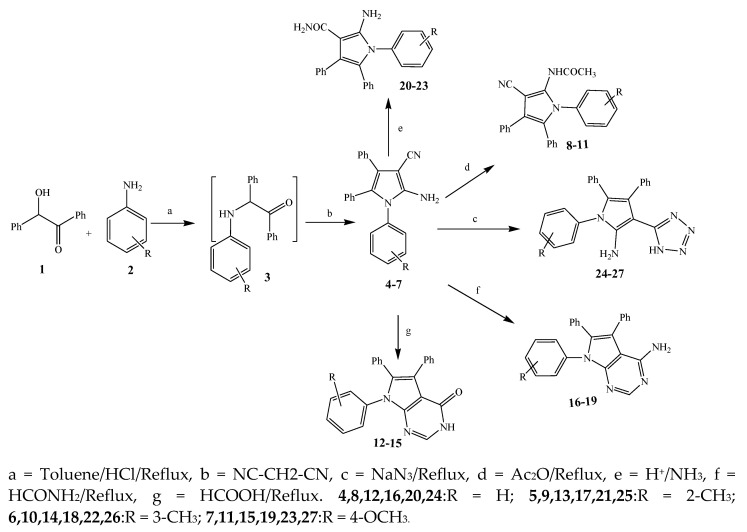
Synthetic route of pyrrole and its derivatives (**4**–**27**).

The synthesis of various derivatives of pyrrole (**4**–**27**) was carried out by Mohamed et al., 2009 from primary aromatic amines (**2**), benzoin (**1**), and malononitrile (Scheme 1). The synthesized compounds were evaluated in terms of their antibacterial activity against *Staphylococcus aureus, Escherichia coli, Bacillus subtilis,* and *Pseudomonas aeruginosa*. Most of the synthesized compounds exhibited excellent activity against these bacterial strains. Some of them showed similar activity while others were two to four times more or less active than the standard drug, amoxicillin. The activity of compounds **6, 10**, and **15** was almost similar to that of the reference drug, whereas **17** was found to be two times more active than amoxicillin against *B. subtilis.* Furthermore, the compound **18** against *S. aureus* displayed activity that was lower than amoxicillin, while **21** and **27** were four times more active than amoxicillin. The compound **5** was also more effective against *E. coli* and **16** showed significant activity against *P. aeruginosa*, which was reciprocal to amoxicillin [16].

**Scheme 2:**
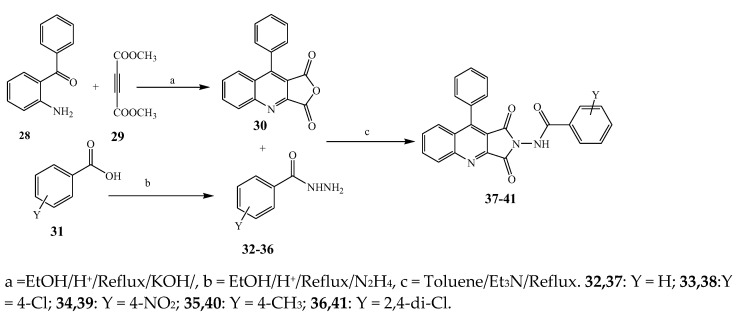
Synthetic route for the synthesis of pyrrole[3,4-b]quinolin-2(3*H*)-yl)bezamide derivatives (**37**–**41**).

**Scheme 3:**
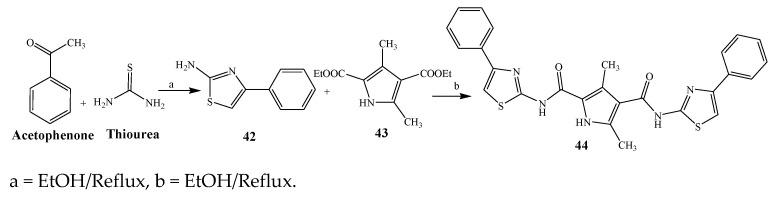
Synthetic route of compound **44**.

**Scheme 4:**
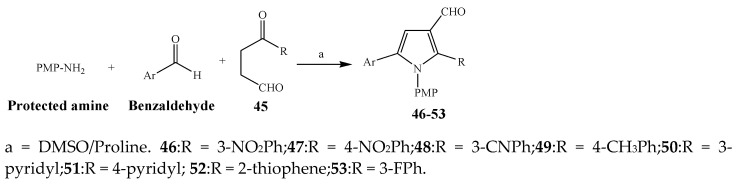
Synthesis of pyrrole from 1,4-dicarbonyls.

**Scheme 5:**
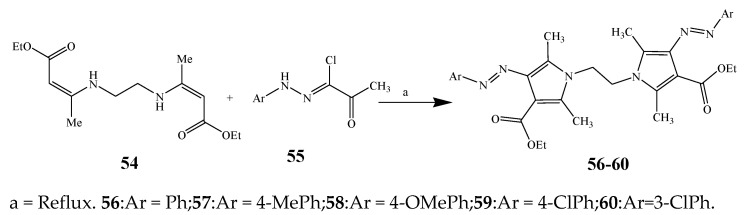
Synthesis of bis-pyrrole derivatives.

**Scheme 6:**
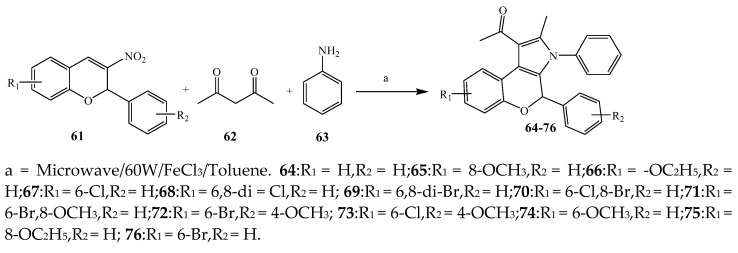
Synthesis of 2*H*-chromene fused pyrrole derivatives (**64**–**76**).

Largani et al., 2017 also synthesized pyrrolo [3,4-b]quinolin-2(3H)-yl)benzamides from benzohydrazide and 9-phenylfuro[3,4-b]quinoline-1,3-dionesas as shown in Scheme 2. The antibacterial activity of compounds **37**–**41** was evaluated against *Staphylococcus aureus*, *E. coli* and *Enterococcus faecalis* which demonstrated vigorous inhibition against these bacterial strains. It was observed that amongst all the targeted compounds, **37** exhibited magnificent activity against *E. faecalis*, *S.* aureus and *E. coli* bacteria with MIC 0.5, 0.25, and 0.25 mg/mL, respectively [19]. The various pyrrole derivatives were prepared (Scheme 3 and Scheme 4) from amino-containing compounds through several steps and their antibacterial activity was evaluated [20]. Compound **42** was synthesized by refluxing acetophenone and thiourea, which was further used for the synthesis of targeted compounds. The synthesized compounds exhibited good antibacterial potential against microbes with an MIC value of 16 µg/mL (Table 1). The activity of compound **44** was equipotent correlated with ciprofloxacin against *S. aureus* and *E. coli* [21]. The protected pyrrole ring was synthesized (**46**–**53**) from **45** by reacting with aromatic aldehyde and protected amine. It was revealed that compounds **50**, **51**, and **52** exhibit valuable antibacterial activity against *Pseudomonas putida* bacterial strains. Compound **49** also showed the equivalent inhibitory potential against the tested strains of bacteria. Similarly, **53** and **49** exhibited remarkable antibacterial activity at the range of 32–64 μg/mL [20]. The synthesis of bis-pyrrole (**56**–**60**) through hydrazonoyl halides (Scheme 5) and their evaluation against four different bacterial strains was reported by Kheder [22]. The synthesized compounds were more active against the positive strains as compared to the negative strains. Furthermore, compound **59** with the Cl group at the *para*-position displayed a high degree of antibacterial activity against *B. subtilis* and *S. aureus*. Similarly, pyrrole derivatives (**64**–**76**) were prepared by a method based on microwave irradiation (Scheme 6) and their antibacterial activity was evaluated against *E. coli* and *S. aureus* [23]. It was also found that the compounds **65** and **71** exhibit significant activity against both *S. aureus* and *E. coli,* while **72** was the least effective. Furthermore, **71** displayed MIC (20 μg/mL in *S. aureus* and *E. coli*) closer to the reference drug, gentamicin. They also explained the SAR of the synthesized compounds, which showed that the substituent 3-formylpyrroles at position C-2 with thiophene (**52**) and pyridine (**50, 51**) had significant antibacterial activity, while 4-pyridyl had a weak response. They further elaborated that the presence of methoxy and ethoxy at positions C-8 and C-6 in the system of chromene increased the efficiency of the compound. The outcome revealed that the substitution at the 2-position of the phenyl ring of the chromene moiety (**72**, **73** and **75**) had the least inhibition of bacterial growth.

### 2.2. Synthesis and Antibacterial Activity of the Pyrimidine

**Scheme 7:**
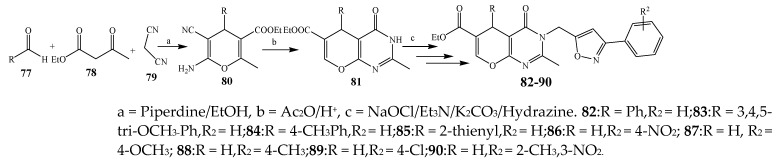
Synthetic route of pyrimidine from aromatic aldehyde (**82**–**90)**.

Pyrano[2,3-*d*]pyrimidine-6-carboxylates (**82**–**90**) were synthesized via a multistep reaction (Scheme 7) [24]. The antibacterial activity for the synthesized compounds was checked against Gram-negative (*Klebsiella pneumonia*, *E. coli*) and Gram-positive (*P. aeruginosa*, *S. aureus*) strains using ciprofloxacin as a standard drug. Compounds **80**–**81**, **83**–**85**, **87**, **89** and **90** exhibited exceptional activity, while compounds **83**, **86,** and **88** showed remarkable activity (Table 1). They further elaborated that compounds with an electron-withdrawing substituent (–OCH_3_ and –NO_2_) exhibited maximum activity as compared to compounds with the methyl group [24].

**Scheme 8:**
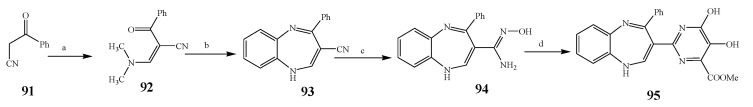
Synthesis of pyrimidine derivatives of 1,5-benzodiazepine from benzoylacetonitrile.

**Scheme 9:**
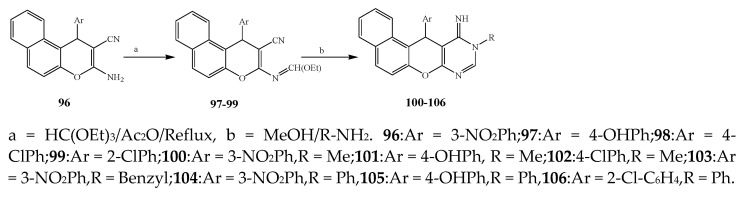
Synthesis of new benzo[5,6]chromeno[2,3-*d*]pyrimidines (**100**–**106**).

**Scheme 10:**
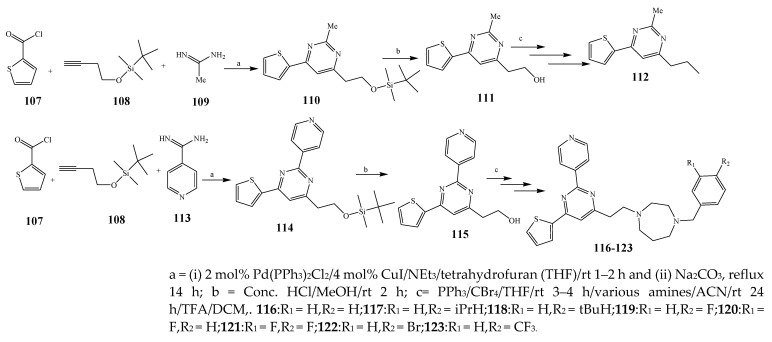
Synthesis of pyrimidine using acetamidine and isonicotinamidine.

**Scheme 11:**
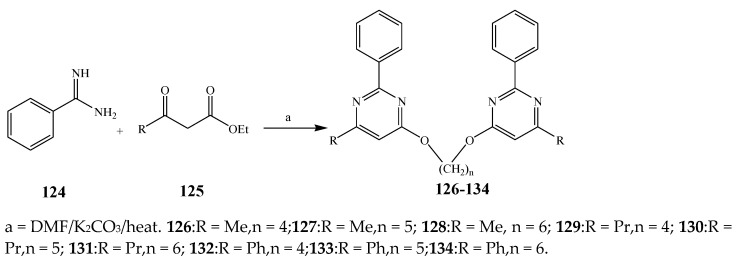
Synthesis of bis-pyrimidines (**126**–**134**).

**Scheme 12:**
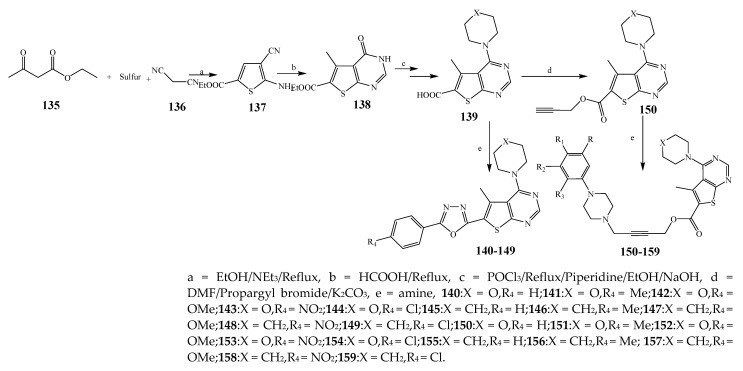
Synthesis of thieno[2,3-*d*]pyrimidine-alkyne.

**Scheme 13:**
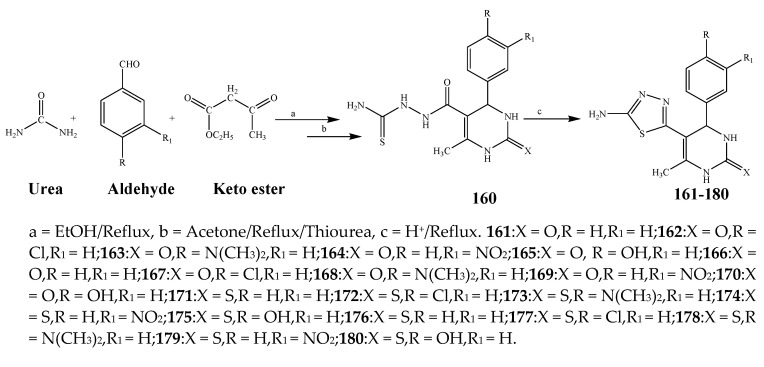
Synthesis of 3,4-dihydro-5-(5-mercapto-4*H*-1,2,4-triazol-3-yl)-6-methyl-4-phenyl pyrimidin-2(1*H*)-one (**161**–**180**).

Misra et al., 2018 reported the synthesis of pyrimidine derivatives with 1,5-benzodiazepines under microwave conditions using 1,4-diazabicyclo[2.2.2]octane and dimethyl acetylenedicarboxylate, as shown in Scheme 8. The antibacterial results indicated that compound **95** had exhibited persuasive inhibitory activity against *E. coli* and *S. aureus* with IC_50_ values of 300 and 200 μg/mL, respectively [25]. The synthesis of (benzo[5,6]chromeno[2,3-*d*]pyrimidine (**96**–**106**) (Scheme 9) was reported by Ameli et al., 2017, while their anti-infectious activity was checked against *S. aureus*, *B. subtilis*, *E. coli* and *Salmonella typhimurium.* Compounds **100**–**106** showed high activity while **96-99** remained moderate (MIC > 250). These results suggested that the pyrimidine moiety plays a significant role in antibacterial activity. Compounds **101**, **102**, **105** and **106** whose aryl groups have a Cl or OH group, displayed greater inhibitory effects against *E. coli*, *S. aureus*, *S. typhimurium* and *B. subtilis* strains which may have the potential to bind the compounds to corresponding receptors [14]. The 2,4-disubstituted-6-thiophenyl-pyrimidine as antibacterial agents was prepared by Fang et al., 2019 and the route of synthesis is presented in Scheme 10. It is estimated that among compounds **116**–**123**, the most potent were **118**, **117** and **123** with 5 μg/mL MIC compared to vancomycin and methicillin (Table 1). On the other hand, the inhibitory effect was weak against *E. coli* which may be due to the lower penetrating capacity of the synthesized compounds to the outer Gram-negative bacterial membrane [26]. The results further showed the presence of the group at the 2nd position of pyrimidine pivotal activity. The cyclo-condensation protocol was also reported for the synthesis of the bis-2-phenylpyrimidines compound from β-ketoester, benzamidine hydrochloride, and dihaloalkanes as shown in Scheme 11 **[27]**. The *in vitro* antibacterial activity of the synthesized compounds **126**–**134** was also studied against *Salmonella enterica* ATCC 13312, *E. coli* ATCC 25922, *Micrococcus luteus* ATCC 29213, *B. subtilis* ATCC 29213, and *S. aureus* ATCC 29213. The results showed that compounds **126**–**134** had good activities towards *B. subtilis*, *S. enterica* and *M. luteus,* while most of the compounds against *S. aureus* showed a weak response [27]. Pyrimidine (**140**–**149**) and oxadiazole (**150**–**159**) were prepared by Triloknadh et al., 2018 as shown in Scheme 12 [28]. Among all the synthesized compounds, **150**, **154**, **155a**, and **156** exhibited vigorous activity with 0.0125 to 0.0412 mg/mL values of MIC, which were higher than gentamicin (MIC = 0.0212 to 0.0422 mg/mL). Compound **141** displayed dominant activity across three strains (*P. aeruginosa*, *B. subtilis* and *S. aureus*) with 0.0124, 0.0120, and 0.0311 mg/mL MIC values, respectively (Table 1). Moreover, if the methoxy group was moved from position 3 to position 4 (**140**), it may lead to the reduction of activity across the strains. In addition, methyl, cyano, and bromo substitutions enhanced the activity. Andrews et al., 2017 described the synthesis of the pyrimidine derivatives (**161**–**170**), (**171**–**180**) as represented in Scheme 13. The *in vitro* antibacterial activity of the recently synthesized compounds was screened against *E. coli*, *S. aureus* and *P. aeruginosa* by agar well disk diffusion technique. The antibacterial activity data showed that compounds **161**–**170** had lower inhibition than compounds **171**–**180** [29].

### 2.3. Synthesis and Antibacterial Activity of the Thiazole

**Scheme 14:**
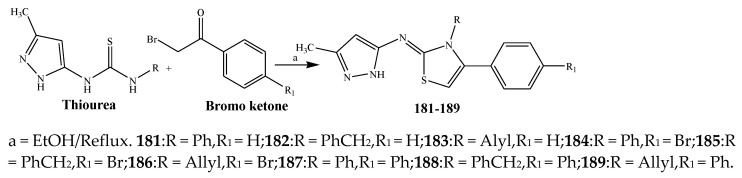
(Thiazol-2-yl)pyrazol-5-amines from pyrazolylthioureas and ω-bromoacetophenones.

**Scheme 15:**
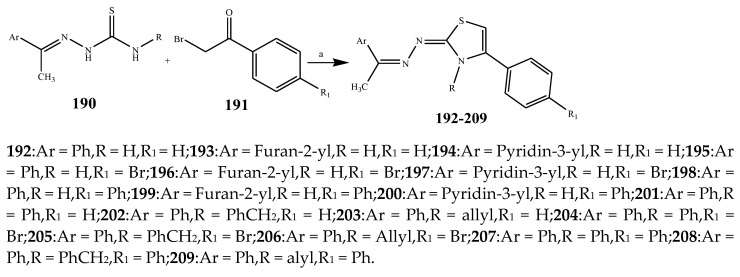
Synthetic route of compounds (**192**–**209**).

**Scheme 16:**
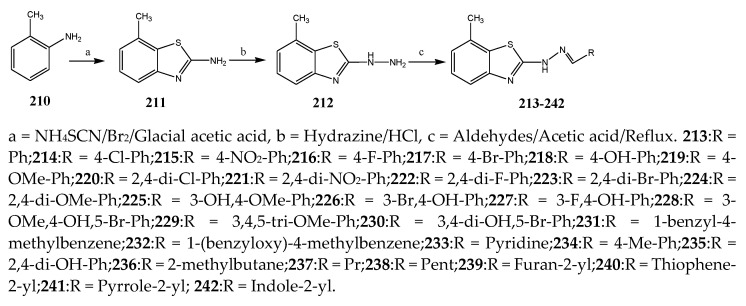
Synthetic routes of various thiazoles.

**Scheme 17:**
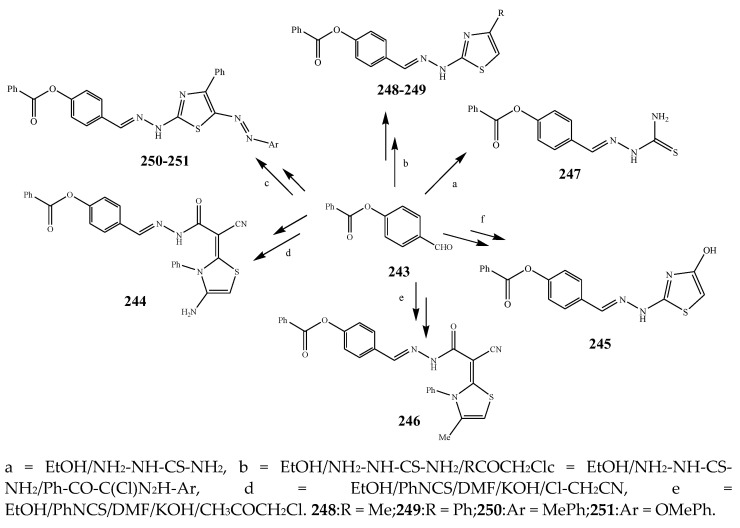
Synthetic routes of thiazole.

**Scheme 18:**
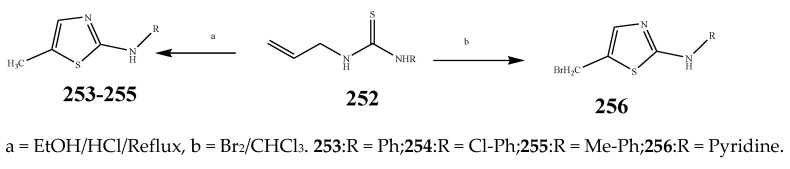
Synthesis of thiazole from thiourea (**253**–**256**).

**Scheme 19:**
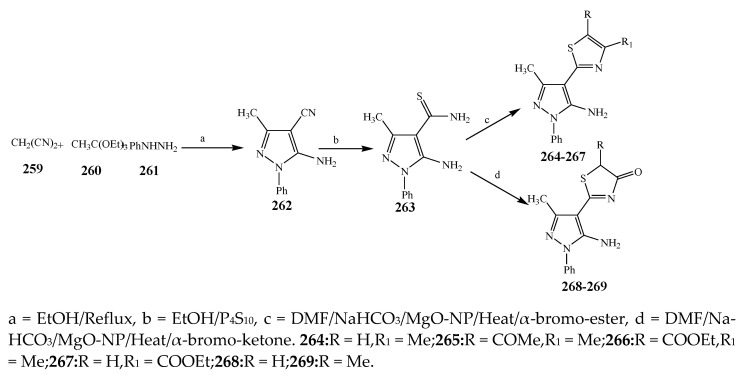
Total synthesis of 4-thiazolylpyrazoles.

**Scheme 20:**
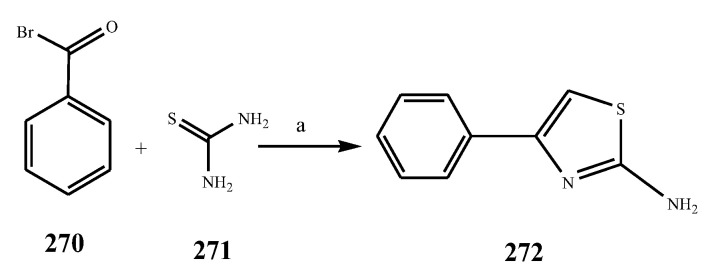
Synthetic routes of substituted thiazole.

**Scheme 21:**
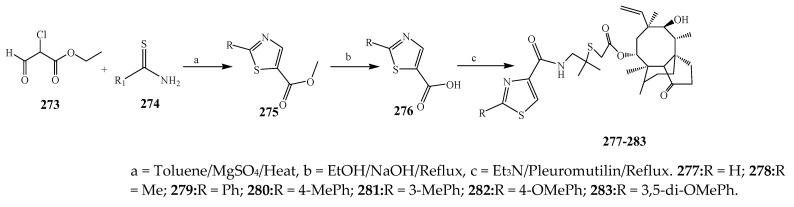
The synthetic route to thiazole having pleuromutilin moiety.

The thiazole derivatives (**181**–**189**), such as 3-substituted-4-aryl-2-[(1-phenylethylidene)hydra-zono]-2,3-dihydrothiazoles and 4-Aryl-2-[2-(1-substituted ethylidene) hydra-zinyl]-thiazoles were prepared through the reaction of bromoacetophenones and (ethylidene)hydrazinecarbothioamides (Scheme 14 and Scheme 15) [30]. Compounds **192**–**209** were evaluated against different Gram-positive and Gram-negative strains of bacteria such as *K. pneumonia*, *P. aeruginosa*, *S. aureus*, and *E. coli* using reference drugs. The results concluded that compounds **192**, **194**, **195**, **200** and **205** had remarkable activity against *E. coli*. Furthermore, compounds **195**, **192**, **196**, **200**, **209** and **207** reported the promising antibacterial activity against *K. pneumonia,* which is greater than ciprofloxacin. Compounds **192**, **194**, **195**, **200**, **205** and **207** exhibited excellent activity against *P. aeruginosa*, which was higher as compared to ciprofloxacin. Moreover, **196**, **200**, **205**, **207** and **209** displayed several folds of activity against *S. aureus.* It was indicated from the results that the existence of a phenyl ring at thiosemicarbazones is necessary for significant activity. Similarly, the allyl, benzyl and phenyl group on the *N*-position of thiazole (**207, 209** and **205**) was associated with a significantly high degree of activity [30]. Thirty thiazole derivatives (**213**–**242**) were synthesized by Zha et al., 2019 as represented in Scheme 16. *In vitro* antibacterial studies of compounds were carried out against *B. subtilis*, *S. aureus*, *K. pneumonia* and *E. coli*. The results explained that compounds **231** and **232** demonstrated valuable activity against all the bacterial strains, while compounds **226** and **227** revealed good activity against *E. coli*, *S. aureus* and *B. subtilis* because of the existence of an electron-donating (-OCH_3_ and -OH) moiety in the molecule [31]. Similarly, Abdel-Galil et al., 2018 reported the synthesis of a series of scaffolds of heterocyclic compounds that contain thiazolidin-5-one and thiazole rings from 4-formylphenyl benzoate precursor (Scheme 17). Antibacterial activity of the synthesized compounds was performed against *S. aureus* and *E. coli*. The results reported that compounds **244**, **245** and **248** exhibited dominant antibacterial activity against the *S. aureus* bacterial strain, while **6** displayed exceptional activity against *E. coli*. Moreover, compounds **251**, **250** and **246** demonstrated acceptable activity across the positive strain and compounds **244** and **247** revealed good activity against the negative strain (Table 1). The existence of ethoxy, methyl, amino, azo, hydroxyl, or methoxy groups assimilated with phenylbenzoate, cyanoacetyl hydrazine, and carbamothioylhydrazone moiety in the derivatives of thiazole depicted excellent antibacterial activity. Furthermore, the presence of an electron-withdrawing group rather than an electron-donating group in compound **249** reduced the activity. The compound **248** showed the excellent antibacterial activity against *S. aureus* and *E. coli* bacterial strains, which may be due to the –CH_3_ group at the 4th position of thiazole [32]. The synthesis of thiazoles from allyl thiourea as given in Scheme 18 was carried out by Khare, et al., 2016. All the synthesized compounds (**253**–**258**) exhibited moderate activity [33]. Beyzaei, et al., 2017 illustrated the synthesis of 4-thiazolylpyrazole (**264**–**269**) compounds from the modified Hantzsch method, in the presence of catalyst MgO nanoparticles as represented in Scheme 19. An evaluation of the *in vitro* antibacterial activity of the synthesized compounds was also performed against 21 bacteria. Compounds **264** to **269** of 4-thiazolylpyrazoles exhibited inhibition activities against *S. agalactiae*, *P. aeruginosa* and *S. pneumoniae*. In comparison with the other compounds, thiazoles **265** and **269** were potent against eight pathogenic bacterial strains, and as such they appeared as one of the effective broad-spectrum agents [34]. Mohamed et al., 2018 reported the synthesis of thiazole derivatives as shown in Scheme 20 and observed that the derivative of **272** had greater antibacterial activity. This may be due to the thiazole’s molecular structure and also the existence of the NH group, which helps the adsorption of a compound onto the surface of bacteria, perforation into their cell membrane, and finally eradication of the membrane, causing the death of bacteria [35]. Seven unique derivatives of pleuromutilin with the moiety of thioether and thiazole-5-carboxamide (**277**–**283**) were prepared by Wang et al., 2011 as represented in Scheme 21. The *in vitro* antibacterial activity of the synthesized compounds was evaluated against two positive strains of bacteria viz. *S. aureus* ATCC26112 and *S. aureus* SC. The compounds exhibited valuable activity against the bacteria and it can be examined that the bacterial strain of *S. aureus* was more susceptible than *S. aureus* [36].

### 2.4. Synthesis and Antibacterial Activity of the Pyridine

**Scheme 22:**
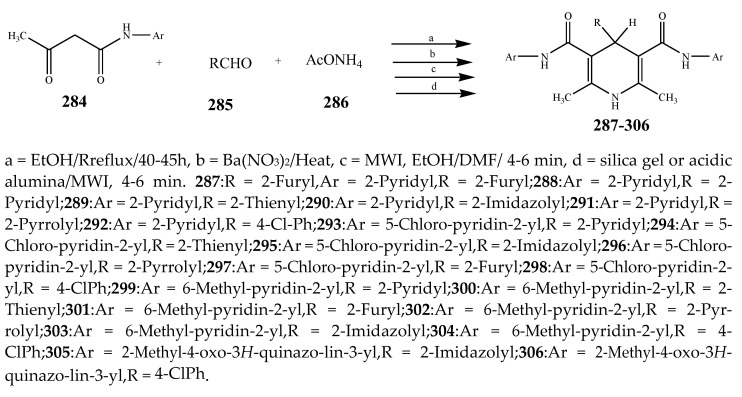
Synthesis of di-substituted pyridine (**287**–**306**).

**Scheme 23:**
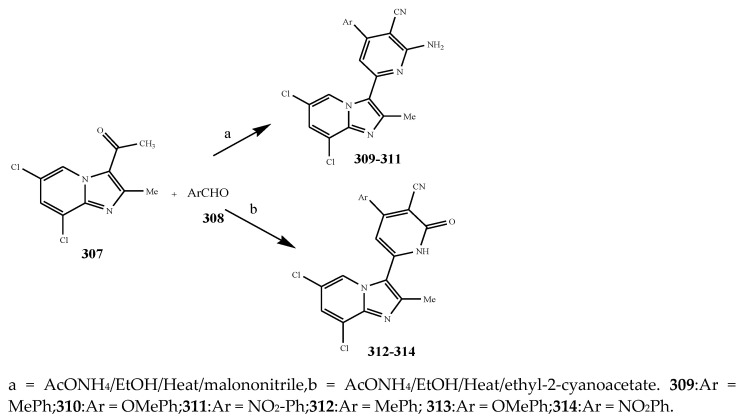
Synthesis of 6,8-dichloro-2-methylimidazo[1,2-*a*]pyridinyl-pyridine hybrids.

**Scheme 24:**
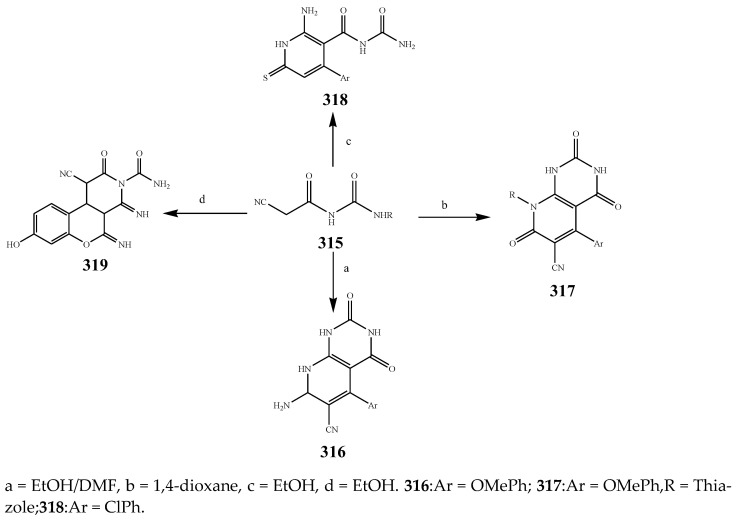
Synthesis of fused heterocyclic compounds (Pyridine).

**Scheme 25:**
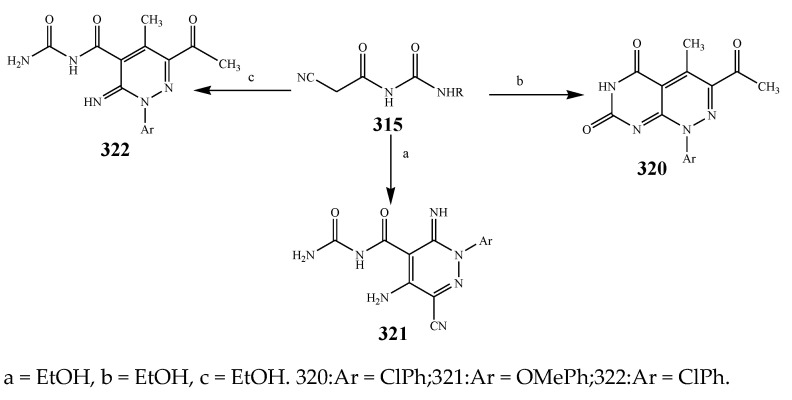
Synthetic route of pyridine from urea derivatives.

**Scheme 26:**
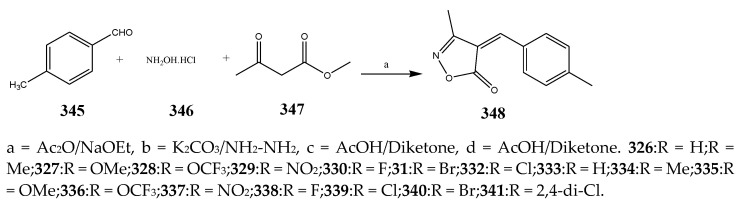
Synthesis of 3-amino-6-(trifluoromethyl)-1*H*-pyrazolo[3,4-*b*] pyridine-5-carboxylate.

**Scheme 27:**
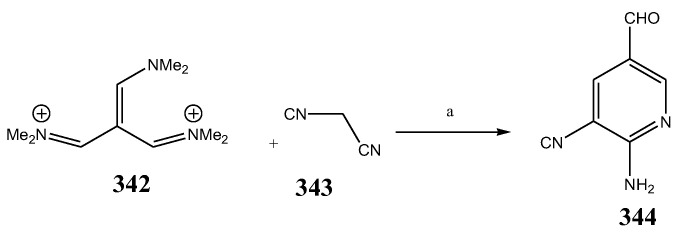
Synthesis of formyl pyridine. a = AcONH_4_/EtOH.

Sirisha, K. et al., 2010 reported the synthesis of twenty 1,4-Dihydropyridines (**287-306**) from a condensation reaction of aromatic aldehyde, ammonium acetate and *N*-heteroaryl acetoacetamide as shown in Scheme 22. The *in vitro* antibacterial activity of the synthesized compounds was executed against the strains using streptomycin and tetracyclin as reference drugs. Data analysis showed that compound **299** with 2-pyridyl at position C-4 and *N*-(6-methylpyridin-2-yl) at the C-5 and C-3 position was equipotent to tetracyclin/streptomycin across negative strains, while compound **305** was more effective against *B. subtilis*. Compounds **292**, **295** and **299** also had good inhibitory activity across MRSA [37]. Althagafi and Latif, 2021 reported the synthesis of the derivatives of 6,8-dichloro-imidazo[1,2-a]pyridine (**309**–**314**), including pyrazole, thiazole, and pyridine ring, as represented in Scheme 23. They evaluated synthesized compounds against *K. pneumonia*, *E. coli*, *B. subtilis*, and *S. aureus*. The antibacterial screening presented that many tested compounds displayed significant activity against both strains of bacteria. The compounds **316**, **317**, **318** and **319** had the maximum inhibitory activity across most of the bacterial species, while **318** exhibited tremendous activity against all strains as compared to gentamycin and ampicillin (Table 1). Furthermore, compounds **317** and **316** revealed significant activity toward *E. coli*. Compound **318** also showed the same value of MIC against *K. pneumonia* [38]. The synthesis of Pyrido[2,3-d]pyrimidine was undertaken by a reaction of the derivatives of cyanoacetylurea with arylidines or aromatic aldehydes as shown in Scheme 24 and Scheme 25. Antibacterial activity was performed for all the targeted compounds against *E. coli* and *S. aureus* strains using gentamicin as a standard drug. From the data of the antibacterial activity, it was observed that the compounds **316**, **320**, **319**, **322** and **321** had displayed tremendous activity across all the strains with a 16.5 to 24mm zone of inhibition (ZOI) value compared to the reference drug (ZOI 15 mm) [39]. Pyrimidine (**326**–**332**) and pyridine (**333**–**341**) were synthesized according to Scheme 26 and screened against different bacteria [40]. The compounds **326**–**332** and **333**–**341** exhibited promising activity against *K. planticola*. Correspondingly, compounds **326**–**332** and **335**–**336** also showed appreciable activity against *S. aureus*. From the SAR study, it was estimated that the activity of the compounds was due to the existence of pyrimidine fused to the skeleton of pyrazolo[3,4-*b*] pyridine with a particular substitution pattern [40]. Pyridine derivatives were synthesized by Jemmezi et al., 2014 from a cyclocondensation reaction as shown in Scheme 27. All the synthesized compounds were screened for their antibacterial activity against *Vibrio vulnificus*, *Vibrio cholera, Vibrio alginoliticus* and *Vibrio parahaemolyticus*. It was revealed that the synthesized compounds had significant biological activities. Compound **344** was found to be the one that showed the best activity, which is comparable to the activity of tetracycline. This was due to the presence of an aldehyde function, which imparted its tremendous biological activities [41].

### 2.5. Synthesis and Antibacterial Activity of the Oxazole

**Scheme 28:**
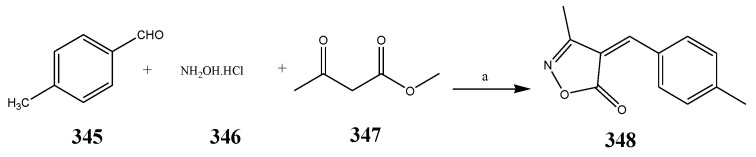
Synthesis of arylmethylene-isoxazol-5(4*H*)-one derivatives. a = LiBr/EtOH.

**Scheme 29:**
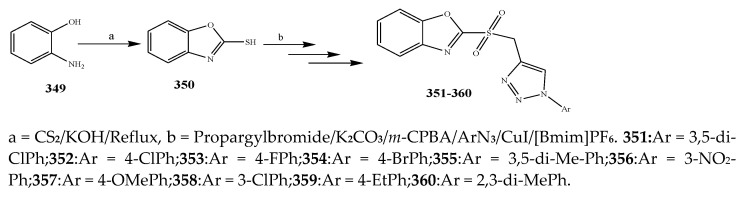
Synthetic route of oxazole from hydroxyl aniline. a = CS_2_/KOH/Reflux, b = Propargylbromide/K_2_CO_3_/*m-*CPBA/ArN_3_/CuI/[Bmim]PF_6_.

**Scheme 30:**
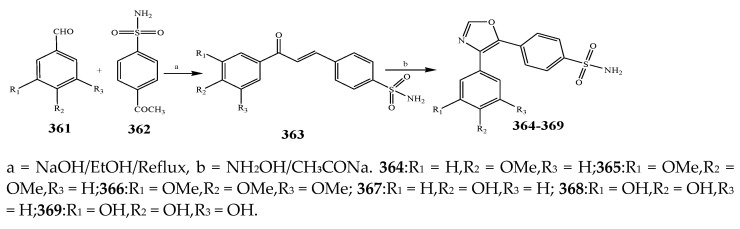
Synthesis of 1,2-oxazoles **364**–**369**.

The synthesis of oxazole was carried out according to the reaction scheme shown in Scheme 28 by Ferouani et al., 2018. The synthesized compounds were evaluated for their in vitro antibacterial activity against *Enterococcus faecium* (DSM 20477), *E. coli* (CECT 515) and *Clostridium perfringens* (CECT 486) using ampicillin and gentamicin as standard drugs. Among the synthesized compounds, the ones with 4-F, 2-OH and 4-CH_3_ exhibited remarkable activity across all three bacterial strains [7]. The compounds also displayed better activity in comparison with the reference drugs, while remaining compounds indicated good activity, except for the one containing 4-N(CH_3_)_2_, which exhibited complete retardation against all the bacterial strains. The compound **348** displayed the equivalent antibacterial activity against *E. faecium* and *E. coli*, while compounds with 4-OMe and 2-F were found to be active across only one bacterial strain each (*C. perfringens* and *E. faecium*, respectively) (Table 1). Babu et al., 2019 reported the synthesis of 2-sulfonyl benzoxazole compounds (**351-360**) comprising the nucleus of 1,2,3-triazole through a highly effective and naturally favorable ionic liquids microwave-assisted method as shown in Scheme 29. Among all the synthesized compounds, **353** with a moiety of 4-fluoro phenyl triazole at 2-sulfonyl benzoxazole showed persuasive activity against *P. aeruginosa* (MIC = 6.25 μg/mL), *E. coli* (MIC = 3.12 μg/mL) and *S. epidermidis* (MIC = 6.25 μg/mL), and mild activity against *B. subtilis* (MIC = 12.5 μg/mL). The other compounds displayed moderate to low activity. All results were analogous to streptomycin [42]. The derivative compounds 1,2-Oxazole (**364**–**369**) were synthesized by Arshad, M. 2018 as shown in Scheme 30. They were estimated computationally to determine their physicochemical properties and also screened for their antibacterial activity against *S. aureus*, *S. epidermidis*, *E. coli*, and *P. mirabilis*. The antibacterial study indicated that four synthesized compounds **365**, **367**, **368** and **369** exhibited activities greater than the reference drug (ciprofloxacin). It was also revealed that all six products displayed larger activity and lesser MIC than the reference drug [43].

**Scheme 31:**
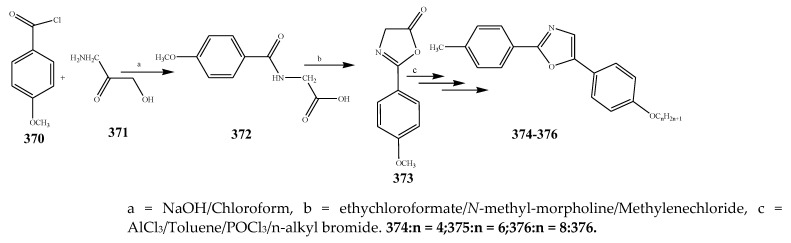
Synthetic route of compounds **374**–**376**.

**Scheme 32:**
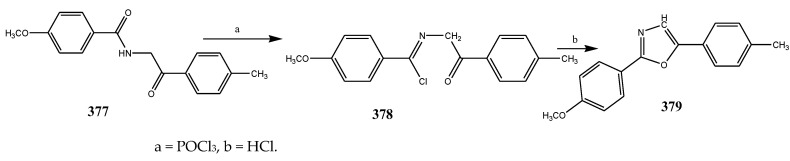
Synthetic route of oxazole (**379**).

**Scheme 33:**
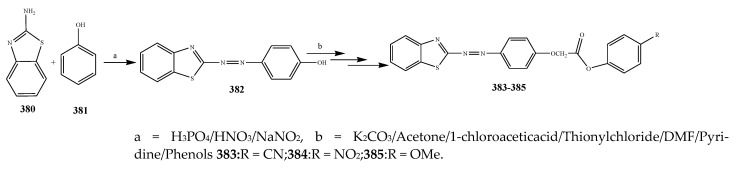
Synthetic route of compounds (**383**–**385**).

**Scheme 34:**
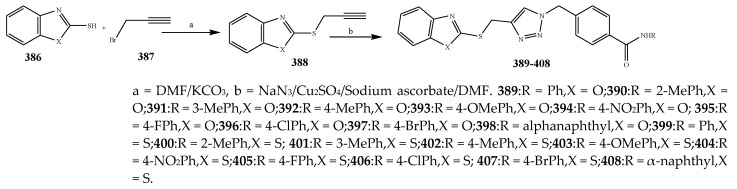
Synthesis of benzothiazole and benzoxazole linked 1,4-disubstituted 1,2,3-triazoles.

**Scheme 35:**
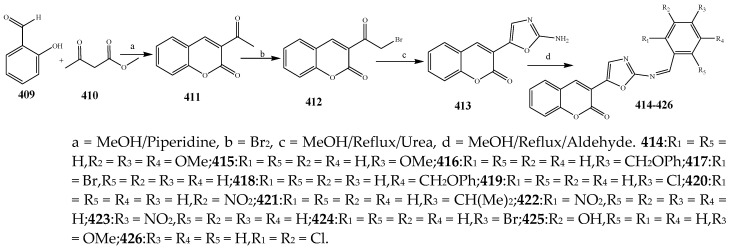
Synthesis of 3-(2-aminooxazol-5-yl)-2*H*-chromen-2-one derivatives (**414**–**426**).

The compounds containing benzothiazole and an oxazole ring were synthesized and evaluated for their antibacterial activity against *P. aerugenosa*, *E. coli*, and *Staphylococcus aureus* using an agar nutrient medium and were compared with ofloxacin by Tomi et al., 2015. The derivatives of benzothiazole **383**–**385** displayed appreciable antibacterial activity against *P. eruginosa*, *E. coli*, and *S. aureus* when correlated with the derivatives of oxazole **374**–**376** (Scheme 31, Scheme 32 and Scheme 33). The compounds **384** and **385** exhibited credible activity against *P. eruginosa* and *E. coli*, respectively, as compared to the reference drug. All the remaining compounds showed mild to valuable antibacterial activity. The derivatives of oxazole **374**–**376** were also recognized to be effective across the strains of bacteria. Compound **376** exhibited higher antibacterial activity towards *P. aerugenosa*, while compounds **374** and **376** showed a similar inhibition value against *E. coli* (Table 1). It was concluded that the derivatives comprising the benzothiazole moiety were more effective than the oxazole moiety [44]. Kaushik et al., 2020 reported the synthesis of benzoxazole and benzothiazole containing the 1,2,3-triazoles moiety (**389**–**408**) through a cycloaddition reaction as represented in Scheme 34. The derivatives were evaluated for their antibacterial activity according to in vitro models against Gram-positive bacteria such as *B. subtilis*, *S. aureus* and Gram-negative strains (*E. coli* and *K. pneumonia*). It was found that in many cases, the existence of any substituent group in the benzene ring enhanced activity against all the verified strains of bacteria. Moreover, the occurrence of an electron-withdrawing moiety in the benzene molecule had displayed a benefit over an electron-donating substituent. Compounds **394** and **402** with the –NO_2_ group exhibited increased activity than its alkyl-substituted or unsubstituted analogs. Improved activity was shown by the 1,2,3-triazoles comprising –CH_3_ group at *m*-position (**391** and **400**) against bacterial strains than its *para* or *ortho* counterparts. Compounds **396** and **407** with the Br group in the benzene ring showed more activity than the compounds with Cl and F substituents. Furthermore, the compounds **397** and **408** comprising the naphthyl ring exhibited potential activity towards all the bacteria. All the derivatives of triazole encompassing the ring of benzothiazole displayed better efficiency in contrast to the ring of benzoxazole [45]. Fifteen compounds (**411**–**426**) with the oxazole ring were prepared by Kakkar et al., 2018 (Scheme 35). They were evaluated against *S. aureus*, *E. coli*, *B. subtilis*, *P. aeruginosa* and *S. enterica* using cefadroxil as a standard drug. The antibacterial outcome showed that compound **416** was moderately effective against *S. aureus* and *E. coli* with MIC 14.8 μM, while compound **421** showed moderate activity against the *B. subtilis* strain of bacteria with MIC value 17.5 μM (Table 1). Moreover, compounds **419** and **425** exhibited MIC 17.8 and 17.3 μM against *S. enteric* and *P. aeruginosa,* respectively. From the SAR study, it was revealed that compounds **421** and **425** have an electron-donating group at *p-*position that enhanced the antibacterial activity against *B. subtilis* and *P. aeruginosa*, respectively. Moreover, the existence of *p*-(phenoxy-methyl)benzene in compound **416** increased the activity against *S. aureus* and *E. coli*. However, the electron-withdrawing moiety (Cl) at *para* position of the compound **419** improved the activity towards *A. niger* and *S. enterica* [46].

### 2.6. Synthesis and Antibacterial Activity of the Thiazolidinone

**Scheme 36:**
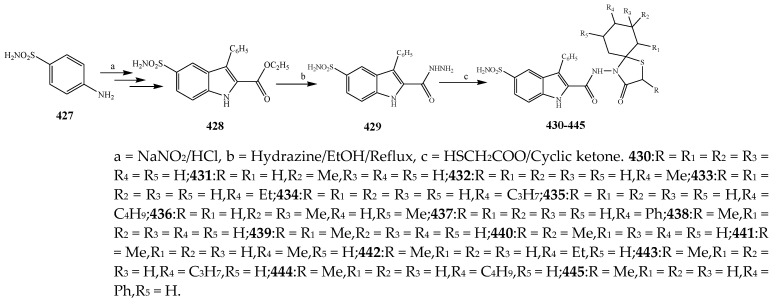
Synthesis of sulfonamide bearing thiazolidinone (**430**–**445**).

**Scheme 37:**
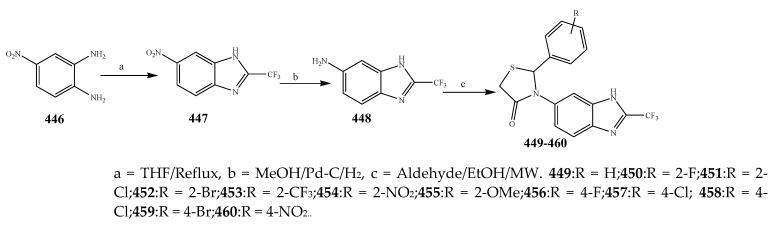
Synthesis of substituted 2-phenyl-3-(2-(trifluoromethyl)-1*H*-benzimidazol-6-yl)thiazolidin-4-ones.

**Scheme 38:**
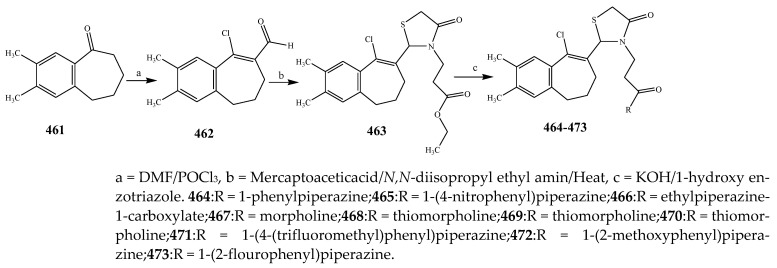
Synthesis of 2-(9-chloro-2,3-dimethyl- 6,7-dihydro-5*H*-benzo[7] annulen-8-yl)-3-(3-oxo-3-[piperazin-1-yl]propyl)thiazolidin-4-one derivatives.

Guzel-Akdemir et al., 2020 reported the synthesis of 4-thiazolidinones and 5-sulfamoyl-1*H*-indoles (**430**–**445**) as represented in Scheme 36 and evaluated the antibacterial activity of the synthesized compounds. Compounds **435** and **444** (with a *tert*-butyl at 8- position on the spirothiazolidinone) and **434** (with propyl moiety at 8-position) showed good activity. The presence of an alkyl substituent at 8-position had stimulated an influence on the activity against *S. epidermidis*. Compounds **440**–**445** exhibited optimum activity against *S. aureus* ATCC 33951, while **440, 442,** and **443** (alkyl groups at 2 and 7 positions) were the most effective against *S. aureus* ATCC 33951 [47]. Cheddie et al., 2020 reported the synthesis of a thiazolidinone (**449**–**460**) derivatives from the *p*-nitro-*o*-phenylenediamine compound as shown in the Scheme 37. The *in vitro* antibacterial activity of the targeted compounds was estimated against two Gram-positive and four Gram-negative strains using levofloxacin and ciprofloxacin as the standard antibiotics. Most of the compounds were 140-fold more effective than levofloxacin and 80-fold more effective than ciprofloxacin against *K. pneumonia* and approximately 140-fold more active than levofloxacin and 600-fold more effective than ciprofloxacin against *S. aureus*. Nitro and Br groups in compounds **452**, **458** and **460** exhibited broad-range activity across all bacterial strains. The 4-Br (**458**) and 2-Br (**452**) derivative compounds indicated excellent activity, greater than that of ciprofloxacin against both the Gram-positive bacterial strains (MRSA = 188.6 μM and *S. aureus* = 94.3 μM). Compound **460** with the NO_2_ group displayed exceptional activity against the Gram-negative strains with MBC 0.15 μM against *E. coli*, *S. typhimurium* and *K. pneumonia* and 9.58 μM against *P. aeruginosa*. It was observed that the phenyl ring has an influence on the activity of the synthesized compounds. Compound **449**, which does not have a Ph substitution, was effective against *S. aureus* and *E. coli* only and had lower activity than the ones with a Ph ring. For instance, the **454** derivative compound with NO_2_ at the 2^nd^ position had reduced activity than compound **460** with NO_2_ at the 4th position against *E. coli* (Table 1). Similarly, compounds containing halogen substitutions on the ring have a profound response toward antibacterial activity. Moreover, benzimidazole compounds with a Ph moiety at C-2, rather than the trifluoromethyl group, also displayed appreciable antimicrobial activity [29]. Some derivative compounds with a thiol moiety at C-2 rather than the trifluoromethyl showed 4-fold better activity against strain-specific bacteria than ciprofloxacin [1]. The thiazolidinone derivatives (**464**–**473**) were also prepared by Jilla et al., 2020 from benzocyclohepetenone as shown in the Scheme 38. The results showed that **469** and **470** displayed significant antibacterial activity against all the strains. Compound **469** showed exceptional activity with MIC 1.9 μg/mL against *B. subtilis* MTCC 121. Compound **470** displayed substantial activity against all the pathogens with MIC 3.8 to 7.9 μg/mL. Compounds **466** and **467** demonstrated promising activity against *P. aeruginosa* MTCC 2453 and *S. aureus* MTCC 96 with 3.9 μg/mL (MIC). In the perspective of the structure–activity relationship, it was noticed that the targeted compounds **469** and **470** possessed secondary amines such as piperidine and pyrrolidine and displayed more probable antimicrobial activity than the compounds with other substituents (Table 1). Furthermore, compounds **464**, **466** and **467,** bearing morpholine, ethyl piperazine-1-carboxylate and phenyl piperazine substituents connected to the benzosuberones compound associated with a scaffold of thiazolidin-4-one, had shown considerable activity correlated with other substituted derivatives [48].

**Scheme 39:**
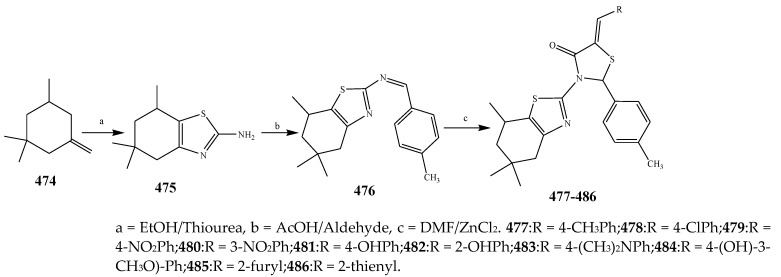
Synthetic pathways for compounds (**477**–**486**).

**Scheme 40:**
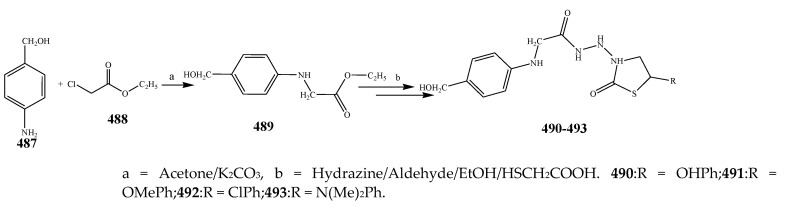
Synthesis of thiazolidinone from benzylalcohol.

**Scheme 41:**
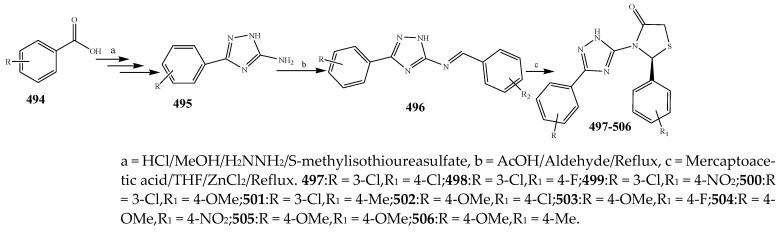
Synthesis of thiazolidinone from benzoic acid (**497**–**506**).

The different derivatives of thiazolidinones (**477**–**486**) were synthesized by the condensation of aryl aldehyde with a basic skeleton as depicted in Scheme 39. The antibacterial activity of all the synthesized compounds was estimated against *P. aeruginosa*, *K. aerogenes*, *C. violaceum B. subtilis*, *S. aureus* and *B. sphaericus* using streptomycin as a standard drug. From antibacterial data, it was revealed that all the synthesized compounds **477**–**486** were active and displayed moderate to valuable activity against bacterial strains (Table 1). Amongst them, compounds **478**, **480** and **486** exhibited excellent antimicrobial activity against *P. aeruginosa,* while compounds **478** and **486** were active against *C. violaceum* [49]. Ismael et al., 2020 reported the synthesis of compounds with pharmacophore of 4-thiazolidinone **490**–**493** as depicted in Scheme 40 and also evaluated their antimicrobial activity against Gram-positive and Gram-negative strains. Compound **492** against *E. coli* exhibited the highest activity which was comparable to that of trimethoprim and also more valuable than the reference drug against *S. Pyougenes* and *Acinetobacter*. In contrast, compound **491** had preferable activity than the standard drug on *S. pyogenes* and was equivalent to the trimethoprim in *Acinetobacter baumanni.* From the comparison of antibacterial results, it was explained that among the tested compounds, compound **492** showed the best activity as compared to **491 [50]**. The 4-thiazolidinones derivatives based on 1,2,4-triazole (**497**–**506**) were reported by Ahmed et al., 2016 (Scheme 41). Antibacterial activity of the synthesized compounds was also evaluated against *P. aeruginosa*, *P. vulgaris* and *E. coli* as Gram-negative and *S. aureus*, *E. faecalis* and *B. subtilis* as Gram-positive strains using moxifloxacin and ciprofloxacin as the control drugs. It was concluded that compounds **502**–**506** exhibited valuable activity with broad-spectrum, while compounds **498**–**501** had not shown any activity across all of the strains. Moreover, it was also found that almost all the tested compounds did not have any activity against *P. vulgaris*. Compound **497** showed reasonable activity against *E. coli* and *S. aureus* with a ~61% zone of inhibition value (Table 1). Most of the derivatives of 1,2,4-triazole exhibited MIC values (8–128 g/mL). The excellent MIC value estimated for the compound **502** was 8 µg/mL against *S. aureus* (Table 1). In the advancement of antibacterial agents, lipophilicity was found to be one of the significant physicochemical parameters, because it was closely associated with permeation through the bacterial lipid layer. The targeted compounds displayed remarkable lipophilic characteristics with 3.4–5.2 *p*-values. From the structural perspective, it appeared that the presence of -OCH_3_, -CH_3_, -NO_2_ and F had auspicious antibacterial activity [51].

### 2.7. Synthesis and Antibacterial Activity of the Imidazole

**Scheme 42:**
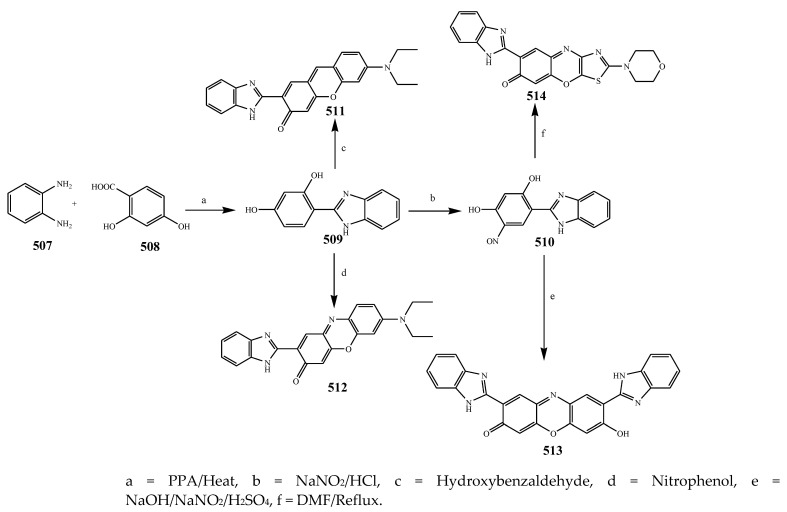
Synthesis of imidazole from diamine.

**Scheme 43:**
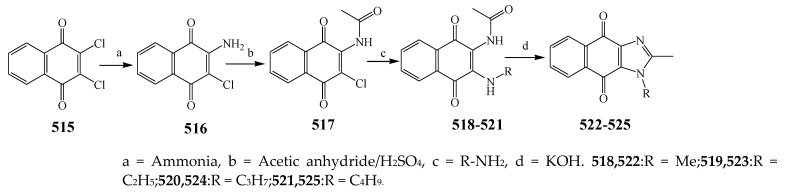
Synthesis of imidazole-based 1,4-naphthoquinone.

**Scheme 44:**
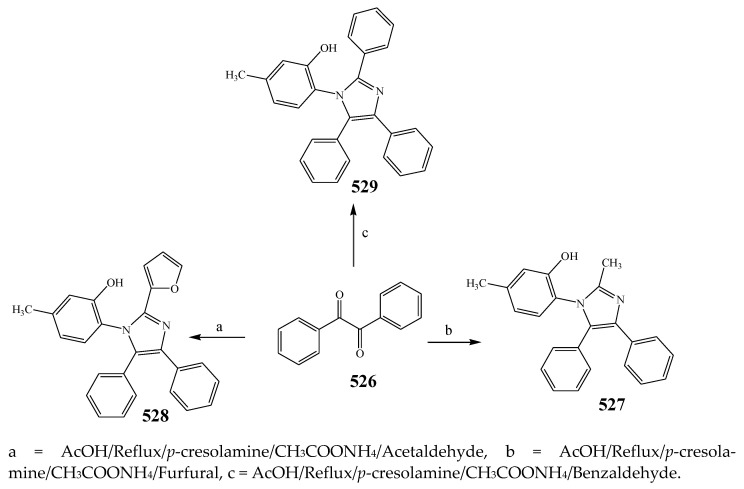
Synthesis of 5-methyl-2-(2-methyl-4,5-diphenyl-1*H*-imidazol-1-yl)phenol**,** 2-[2-(furan-2-yl)-4,5-diphenyl-1*H*-imidazol-1-yl]-5- methylphenol and 5-methyl-2-(2, 4, 5-triphenyl-1*H*-imidazole-1-yl) phenol.

The synthesis and antibacterial evaluation of the different derivatives of the imidazole was undertaken by Patil et al., 2015 as shown in Scheme 42**.** *S. aureus*, *B. subtilis*, *E. coli* and *Micrococcus* were used in biological studies, while sulfamethoxazole and ciprofloxacin were used as the standard drugs. Among the four synthesized compounds, **514** showed the highest activity against all strains. Furthermore, compounds **511** and **512** with electron-donating groups depicted good activity against *Micrococcus*, *E. coli* and *S. aureus*, but slightly less activity toward *B. subtilis*. On the other hand, compound **513** substituted with benzimidazole as an electron-withdrawing group displayed equivalent activity against *Micrococcus*, *E. coli* and *S. aureus* and comparatively remained inactive against *B. subtilis* [52]. Choudhari et al., 2020 reported the synthesis of imidazole compounds based on 1,4-naphthoquinones (**518**–**521**) and (**522**–**525**) as presented in Scheme 43. All the synthesized compounds displayed a wide spectrum of antibacterial activity towards all strains. Some compounds showed MIC values that were comparably smaller than the reference drugs. The compounds also exhibited significant MIC towards isolates of bacteria viz. *P. aeruginosa* NCIM 5029, *E. coli* NCIM 2931 and *S. aureus* 5021. Furthermore, the reference antibiotics exhibited a relatively greater MIC value against both strains of *Pseudomonas*. Compounds **516** and **517** were found to be the 1st and 2nd most active compounds in comparison with the remaining synthesized compounds in terms of MIC. Compound **518** showed a higher MIC value, in the range of 128–512 μg/mL, against all strains (Table 1). Compounds **519** and **520** exhibited MIC = 32 and 64 μg/mL, respectively, against *P. aeruginosa* NCIM 5029. Compound **521** showed an MIC value of 32μg/mL against P*. aeruginosa* NCIM 5029, 64 μg/mL against *P. vulgaris* NCIM 2027 and *S. aureus* NCIM 2079. Compound **525** displayed the best activity against *S. aureus* NCIM 5021 with an 8 μg/mL MIC, while 128 μg/mL MIC was observed against the remaining strains. Compound **524** displayed activity with 8 μg/mL MIC against *S. aureus* NCIM 5021, whereas a 16 μg/mL value against *P. aeruginosa* NCIM 5029 and 128 μg/mL value against *S. aureus* NCIM 2079 were found. Compounds **522**–**525** demonstrated favorable antibacterial activity towards *S. aureus* with 8 μg/mL MIC, but the good value was noticed for *E. coli* and *S. aureus* [53]. Shahzad et al., 2020 illustrated the formation of a tetra-substituted imidazole class of compounds (**527, 528** and **529**) by a cyclocondensation reaction of aromatic primary amines, benzil, aldehydes and C_2_H_7_NO_2_ as represented in Scheme 44. Synthesized compounds were also evaluated for their antibacterial activity against two *S. aureus*, *B. subtilis* and *E. coli* using ciprofloxacin as a standard drug. Compound **528** was found to have tremendous efficacy against bacterial strains. Furthermore, the compound **527** showed the lowest activity (Table 1). Compounds **528** and **529** displayed promising results (>20 mm) against all bacterial species. Moreover, compound **528** showed maximum activity against *B. subtilis* among the three compounds (23 mm) [54].

**Table 1:** Compounds with good antibacterial activities.

Heterocyclic Compounds	Number/Code	Microbes	Reference
**Pyrrole**	**6**, **10**, **15** and **17**	*B. subtilis*	[16]
	**16**	*P. aeruginosa*	[16]
	**18**	*S. aureus*	[16]
	**5**	*E. coli*	[16]
	**37**	*E. faecalis*, *S. aureus*, and *E. coli*	[19]
	**44**	*S. aureus* and *E. coli*	[21]
	**50**, **51**, and **52**	*P. putida*	[20]
	**65** and **71**	*S. aureus* and *E. coli*	[22]
	**49**	*B. subtilis* and *S. aureus*	[23]
**Pyrimidine**	**82**, **86**, and **88**	*P. aeruginosa*, *S. aureus*	[24]
	**95**	*E. coli* and *S. aureus*	[25]
	**102**, **101** and **106**	*S. aureus*, *B. subtilis*, *E. coli*, and *S. typhimurium*	[14]
	**118**, **117**, and **123**	*E. coli*	[26]
	**126** **–** **134**	*B. subtilis*, *S. enterica*, and *M. luteus*	[27]
	**141**	*P. aeruginosa*, *B. subtilis*, *S. aureus*	[28]
	**161** **–** **170**	*E. coli*, *S. aureus*, and *P. aeruginosa*	[29]
**Thiazole**	**192**, **195**, **194**, **200** and **205**	*E. coli*	[30]
	**195**, **200**, **192**, **209**, and **207**	*K. pneumonia*	[30]
	**194**, **200**, **192**, **207**, and **205**	*P. aeruginosa* higher	[30]
	**200**, **205**, **209**, **207**, and **196**	*S. aureus*	[30]
	**226** and **227**	*E. coli*, *S. aureus*, and *B. subtilis*	[31]
	**244**, **247**, **247**, and **248**	*S. aureus*	[32]
	**247**	*E. coli*	[32]
	**248**	*S. aureus* and *E. coli*	[32]
**Pyridine**	**305**	*B. subtilis*	[37]
	**291**	*B. subtilis* and *S. aureus*	[38]
	**317** and **316**	*E. coli*	[38]
	**318**	*K. pneumonia*	[38]
	**326–332** and **333–341**	*Klebsiella planticola*	[40]
	**344**	*Vibrio parahaemolyticus*	[41]
**Oxazole**	**348**	*E. faecium*, and *E. coli*	[7]
	**353**	*P. aeruginosa*	[42]
	**365**, **367**, **368**, and **369**	*S. epidermidis*, *E. coli*, and *P. mirabilis*	[43]
	**385**	*P. aerugenosa*	[44]
	**384**	*E. coli*	[44]
	**376**	*P. aerugenosa*	[44]
	**374**	*E. coli*	[44]
	**394** and **402**	*E. coli* and *K. pneumonia*	[45]
	**416**	*S. aureus* and *E. coli*	[46]
	**419**	*S. enteric*	[46]
**Thiazolidinone**	**434**	*S. epidermidis*	[47]
	**458**	*S. aureus*	[29]
	**460**	*E. coli*	[29]
	**469**	*B. subtilis*	[48]
	**466**	*P. aeruginosa*	[48]
	**478** and **486**	*C. violaceum*	[49]
	**479**	*E. Coli*	[50]
	**497**	*E. coli* and *S. aureus*	[51]
	**502**	*S. aureus*	[51]
**Imidazole**	**518**	*P. aeruginosa* and *E. coli*	[53]
	**527**	*B. subtilis*	[54]

## 3. Conclusions and Future Perspectives

With the increasing population on the earth, health issues are becoming alarming for chemists/pharmaceutics. As microbes become resistant against the available drugs in the market, there is a need for new strategies or routes for the designing or synthesis of new drugs that may be more effective against such microbes. In this review, the data of various heterocyclic compounds with antibacterial activities were narrated from various research groups. Most of the derivative compounds exhibited exceptional to moderate antibacterial activity. There were some factors from a biological perspective, due to which most of the compounds exhibited weaknesses such as: (a) it was difficult for most of the targeted compound to pass through the bacterial membranous wall; (b) specific enzyme production in bacteria which wipe out the drug compound; (c) most of the structural parts had altered the bacteria itself, so the particular drug compound had shown their effect on it. Here, we discussed detailed information on the antibacterial activity of various heterocyclic compounds. It is evident from different studies that the presence of different substituents has a diverse effect, as the electron-withdrawing substitution in the benzene ring enhances the activity as compared to the electron-donating substituent. As heterocyclic compounds have an immense application in the medicinal field, there is a need for more work on synthesis, as well as on their evolution in terms of infectious diseases. Thus, this piece of work would be helpful for the synthesis of new and effective drug molecules.

## Data Availability

Not applicable.
